# Yeast Cth2 protein represses the translation of ARE-containing mRNAs in response to iron deficiency

**DOI:** 10.1371/journal.pgen.1007476

**Published:** 2018-06-18

**Authors:** Lucía Ramos-Alonso, Antonia María Romero, Maria Àngel Soler, Ana Perea-García, Paula Alepuz, Sergi Puig, María Teresa Martínez-Pastor

**Affiliations:** 1 Departamento de Biotecnología, Instituto de Agroquímica y Tecnología de Alimentos (IATA), Consejo Superior de Investigaciones Científicas (CSIC), Paterna, Valencia, Spain; 2 Departamento de Bioquímica y Biología Molecular, Universitat de València, Valencia, Spain; 3 ERI Biotecmed, Universitat de València, Burjassot, Valencia, Spain; University of Surrey, UNITED KINGDOM

## Abstract

In response to iron deficiency, the budding yeast *Saccharomyces cerevisiae* undergoes a metabolic remodeling in order to optimize iron utilization. The tandem zinc finger (TZF)-containing protein Cth2 plays a critical role in this adaptation by binding and promoting the degradation of multiple mRNAs that contain AU-rich elements (AREs). Here, we demonstrate that Cth2 also functions as a translational repressor of its target mRNAs. By complementary approaches, we demonstrate that Cth2 protein inhibits the translation of *SDH4*, which encodes a subunit of succinate dehydrogenase, and *CTH2* mRNAs in response to iron depletion. Both the AREs within *SDH4* and *CTH2* transcripts, and the Cth2 TZF are essential for translational repression. We show that the role played by Cth2 as a negative translational regulator extends to other mRNA targets such as *WTM1*, *CCP1* and *HEM15*. A structure-function analysis of Cth2 protein suggests that the Cth2 amino-terminal domain (NTD) is important for both mRNA turnover and translation inhibition, while its carboxy-terminal domain (CTD) only participates in the regulation of translation, but is dispensable for mRNA degradation. Finally, we demonstrate that the Cth2 CTD is physiologically relevant for adaptation to iron deficiency.

## Author summary

Iron is essential for eukaryotes because it is required for many fundamental processes such as DNA replication, protein translation or respiration, but it is very insoluble and can, therefore, easily go scarce. For this reason, eukaryotic cells have developed adaptive responses to iron deficiency. Under iron limitation conditions, the yeast *Saccharomyces cerevisiae* induces the expression of Cth2, a protein with tandem zinc fingers that binds to adenine and uracil-rich sequences in the 3’-UTR of specific mRNAs related to iron metabolism, promoting their degradation. Here we show that Cth2 inhibits the translation of ARE-containing mRNAs, including *SDH4*, *WTM1*, *HEM15* and *CCP1*, which encode proteins that contain iron or participate in iron-dependent pathways, and *CTH2* itself, which is subjected to an autoregulatory loop that controls its expression. We also dissected different domains of Cth2 that are differentially involved in mRNA decay and translational inhibition. The involvement of Cth2 in translational control reinforces the importance of this ARE-binding protein as a post-transcriptional regulator of the iron response in yeast. By acting at different steps in the life of specific mRNA targets, Cth2 action ensures yeast cells a proper distribution of iron by optimizing its utilization in essential processes.

## Introduction

Iron is indispensable for all eukaryotic organisms because it serves as a redox cofactor in a wide range of critical biological processes, including the synthesis of the principal cellular components (DNA, proteins, lipids and other metabolites), mitochondrial respiration, photosynthesis, and oxygen sensing and transport. Despite being very abundant, iron bioavailability is extremely low. Consequently, cells have developed refined transcriptional and post-transcriptional strategies to properly respond to iron depletion. In response to iron deficiency, the budding yeast *Saccharomyces cerevisiae* activates the expression of Cth2, an RNA-binding protein that, in coordination with its partially redundant homolog Cth1, promotes the metabolic remodeling of cellular processes in order to optimize iron utilization [1,2]. Cth1 and Cth2 belong to a family of proteins whose most studied exponent is mammalian tristetraprolin (TTP/Tis11) [3–8]. The most remarkable trait of the TTP family of proteins is the presence of two highly conserved Cx_8_Cx_5_Cx_3_H tandem zinc fingers (TZFs) through which they specifically bind to AU-rich elements (AREs) within the 3' untranslated region (3'-UTR) of many target mRNAs. The main function assigned to TTP-family proteins consists in promoting ARE-mediated mRNA decay (AMD).

Yeast Cth2 protein shuttles between the nucleus and the cytoplasm, and performs different ARE-dependent functions according to its subcellular localization. The binding of Cth2 to ARE-containing mRNAs takes place in the nucleus, where it can promote the degradation of extended transcripts by influencing the 3ʼ-end processing of its target mRNAs [9,10]. Mature Cth2-bound mRNAs are then translocated to the cytosol through mRNA export pathways and directed to specific sites to undergo degradation via the RNA helicase Dhh1 and the 5ʼ to 3ʼ exonuclease Xrn1 [11]. Upon iron scarcity, Cth2 promotes the degradation of multiple ARE-containing mRNAs including those that encode a subunit of succinate dehydrogenase (*SDH4*), ferrochelatase (*HEM15*), mitochondrial cytochrome-c peroxidase (*CCP1*) and a ribonucleotide reductase inhibitor (*WTM1*) [1,12]. The Cth2 protein contains three regions (CR1, CR2 and CR3) conserved in the *Saccharomyces* species [9]. Previous studies have demonstrated that CR1 is important for the mechanism of *SDH4* mRNA decay, but not for nucleocytoplasmic shuttling [9,13]. Besides, the Cth2 protein contains an ARE within the 3’-UTR of its mRNA that facilitates its auto-degradation [13]. Cth2 negative feedback regulation is important to rapidly recover mitochondrial respiration and growth when yeast cells shift from iron-deficient to iron-sufficient conditions [13].

Mammalian TTP proteins have been implicated in the regulation of diverse physiological processes including inflammatory and immune responses. Interestingly, recent studies have also implicated mammalian TTP protein in the regulation of iron metabolism by promoting the turnover of mRNAs that encode for iron-containing proteins, such as Transferrin receptor 1 [14–16]. TTP protein interacts with multiple components of the mRNA decay and translation repression machineries, including the CCR4/CAF1/NOT1 complex, cap-binding factor eIF4E2, the Dhh1 homolog RCK/p54, the poly(A)-binding protein and the exosome [17–23]. Thus TTP-dependent AMD can proceed through the Xrn1-dependent 5’ to 3’ degradation pathway and via the exosome in a 3’ to 5’ mRNA decay mechanism [18,20,24]. Furthermore, in addition to promote AMD, mammalian TTP represses translation through interactions with RCK/p54, which acts as both a promoter of decapping and a translational repressor [17,25]. Like yeast Cth2, mammalian TTP-family proteins also shuttle between the nucleus and the cytoplasm, and regulate mRNA biogenesis and fate at many levels [5,26–28]. Moreover, the TTP-family member Tis11b modulates the expression of *DII4* mRNA in endothelial cells by interfering with its habitual 3’-end processing [29]. Similarly to its yeast counterparts, feedback regulatory loops have been described for TTP, in this case at both the mRNA decay and translational levels [25,30–32].

We previously reported that yeast cells which express an ARE mutant allele of the *CTH2* transcript display a poor correlation between *CTH2* mRNA and Cth2 protein levels [13]. These observations prompted us to investigate a potential role for Cth2 in targeted translational regulation. Here we demonstrate a new mRNA regulatory function for Cth2 as an inhibitor of the translation of specific transcripts under iron-deficient conditions. The Cth2 function in mRNA translation regulation relies on the presence of an ARE within the target mRNA and an intact TZF domain in the Cth2 protein. Finally, we performed a Cth2 structure-function analysis to decipher the contribution of each conserved domain to either mRNA decay or translation regulation.

## Results

### *SDH4* mRNA translation decreases under iron deficiency

**Fig 1:**
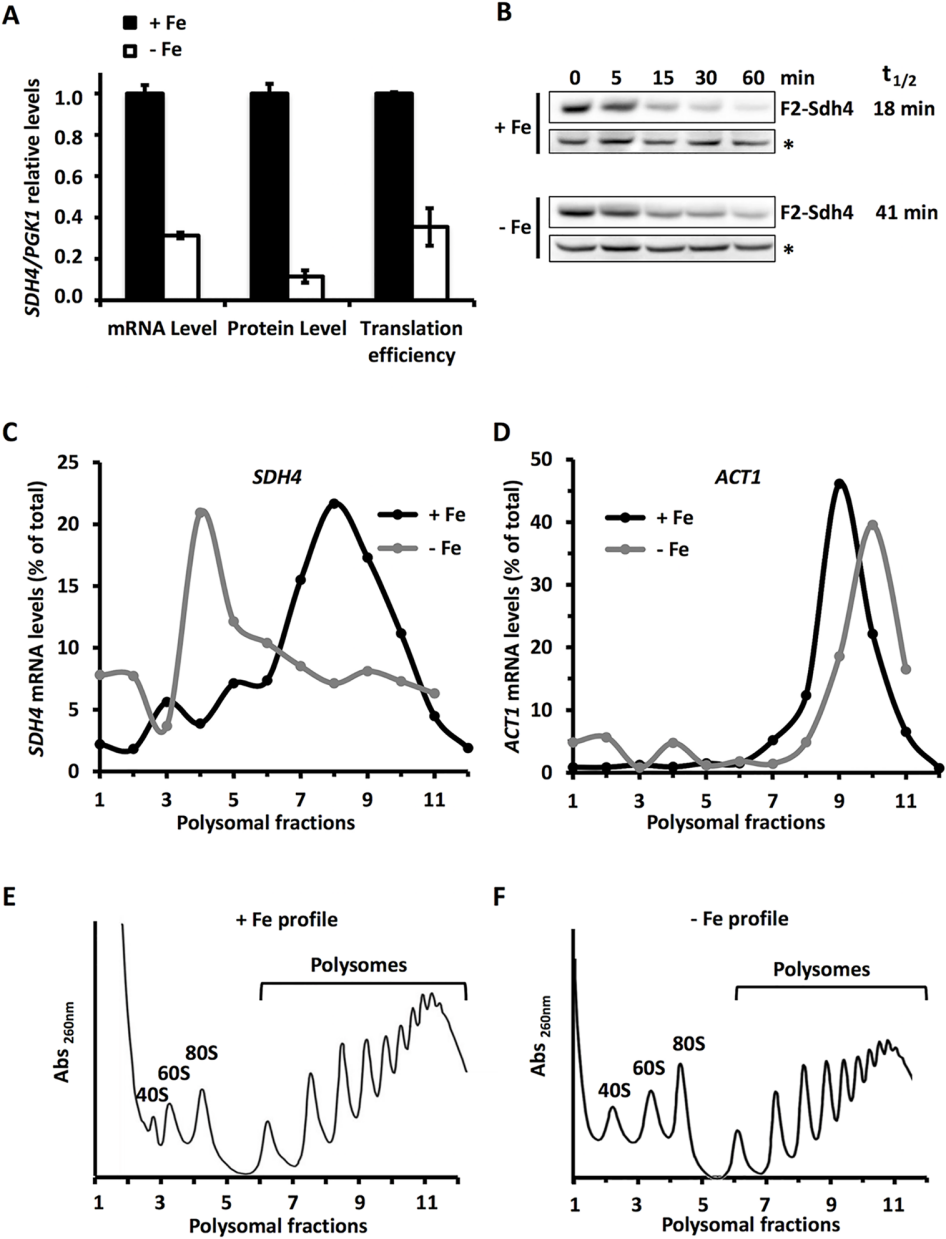
The *SDH4* mRNA is translationally repressed under iron deficiency. **(A)** Wild-type BY4741 strain transformed with pRS416-*Flag*_*2*_*-SDH4* (*SDH4*) was grown at 30°C for 7 h in SC-Ura with 10 μM FAS (+Fe) or in SC-Ura with 100 μM BPS (-Fe). *Flag*_*2*_*-SDH4* and *PGK1* mRNA levels were determined by RT-qPCR using specific primers. Flag_2_-Sdh4 and Pgk1 protein levels were determined by Western blot with anti-Flag and anti-Pgk1 antibodies, respectively. Flag_2_-*SDH4* translation efficiency was calculated as follows: (Flag_2_-Sdh4 protein / *Flag*_*2*_-*SDH4* mRNA) / (Pgk1 protein / *PGK1* mRNA). Mean values and standard deviations from at least two independent experiments are shown and referred to those in +Fe. **(B)** Flag_2_-Sdh4 (F2-Sdh4)-expressing cells were grown as in (A) and Flag_2_-Sdh4 protein levels were determined at successive times after adding 50 μg/mL of CHX. A representative experiment is shown. Mean values of F2-Sdh4 protein half-life (t_1/2_) from two independent experiments are shown. A non-specific anti-Flag band (*) was used as loading control. **(C, D, E, and F)** *sdh4Δ* mutant strain transformed with pRS416-SDH4 plasmid (*SDH4*) was cultivated as mentioned above and polysomal fractionation was carried out as described in Materials and Methods. The A_260nm_ profiles after gradient fractionation in +Fe and -Fe are shown (E and F, respectively), and the ribosomal subunits (40S and 60S), monosomes (80S) and polysomes are indicated. The RNA in individual fractions was extracted and *SDH4* (C) and *ACT1* (D) mRNA levels were analyzed by RT-qPCR as described in Materials and Methods.

We have previously reported that the mRNA levels of *SDH4*, which encodes a subunit of succinate dehydrogenase, lower in response to iron starvation in a Cth2- and ARE-dependent manner [1]. Given that TTP limits the expression of its target mRNAs, not only by promoting their degradation but also by inhibiting their translation, we decided to investigate whether the down-regulation of *SDH4* expression by iron depletion could also be controlled at the translational level. Therefore, we analyzed the translation stage of *SDH4* mRNAs in yeast cells grown under iron-sufficient (SC medium supplemented with 10 μM FAS, +Fe) and iron-deficient (SC supplemented with 100 μM BPS, -Fe) conditions. Translation was measured by two different approaches. First, we defined *SDH4* translation efficiency as the Sdh4 protein/*SDH4* mRNA ratio, normalized to the translation efficiency of 3-phosphoglycerate kinase *PGK1*. For protein level determinations, we did Western blot analyses of a Flag epitope-tagged version of Sdh4 protein and specific Pgk1 antibodies. To assess the mRNA levels, we used RT-qPCR with specific *Flag*_*2*_*-SDH4* and *PGK1* oligonucleotides (see S1 Table). As expected, iron depletion led to a drop in *SDH4* mRNA to 31% of the iron-sufficiency levels (Fig 1A). However, the Flag_2_-Sdh4 protein levels lowered to a greater extent (11% of iron-sufficiency levels). Neither *PGK1* mRNA not Pgk1 protein levels were altered by changes in iron availability. These results indicated that Sdh4 translation efficiency defined as (Sdh4/*SDH4*)/(Pgk1/*PGK1*) decreased 3-fold under iron-deficient conditions (Fig 1A). However, the enhanced reduction of the Sdh4 protein levels observed in iron deficiency could also be explained by a diminished Sdh4 protein stability. This alternative possibility impelled us to analyze the half-life of the Flag_2_-Sdh4 protein under iron-sufficient and iron-deficient conditions by using cycloheximide (CHX) as an inhibitor of translation. As shown in Fig 1B, under low iron conditions (-Fe) the Flag_2_-Sdh4 protein half-life (~41 min) was approximately twice as long as the half-life obtained in high iron conditions (+Fe: ~18 min). Therefore, the drop in the Sdh4 protein levels observed under low iron conditions was not due to Sdh4 destabilization. Instead by considering Sdh4 protein stability, we could conclude that Sdh4 translation efficiency decreases by 6-fold upon iron deficiency. To further test the *SDH4* translational levels, we followed a second experimental approach. We obtained polysome profiles under high and low iron conditions, and then we determined the distribution of *SDH4* mRNA among the different polysomal fractions. Our results showed that, under iron-sufficient conditions, *SDH4* mRNAs were greatly associated with the heavy polysomal fractions, whereas *SDH4* mRNA abundance shifted to the monosomal 80S peak under iron depletion (Fig 1C and 1E). On the contrary, the polysome profile of the actin *ACT1* mRNA, which was used as a non iron regulated control, was seen to be highly associated with polyribosomes under both +Fe and -Fe conditions (Fig 1D and 1F). These results indicated that the association of multiple ribosomes to *SDH4* mRNA, but not *ACT1* mRNA, greatly diminished under iron-limited conditions. Altogether, these two experimental approaches strongly suggest that *SDH4* mRNA is translationally inhibited under iron deficiency.

### AREs within *SDH4* mRNA are essential for its translation inhibition by iron deprivation

**Fig 2:**
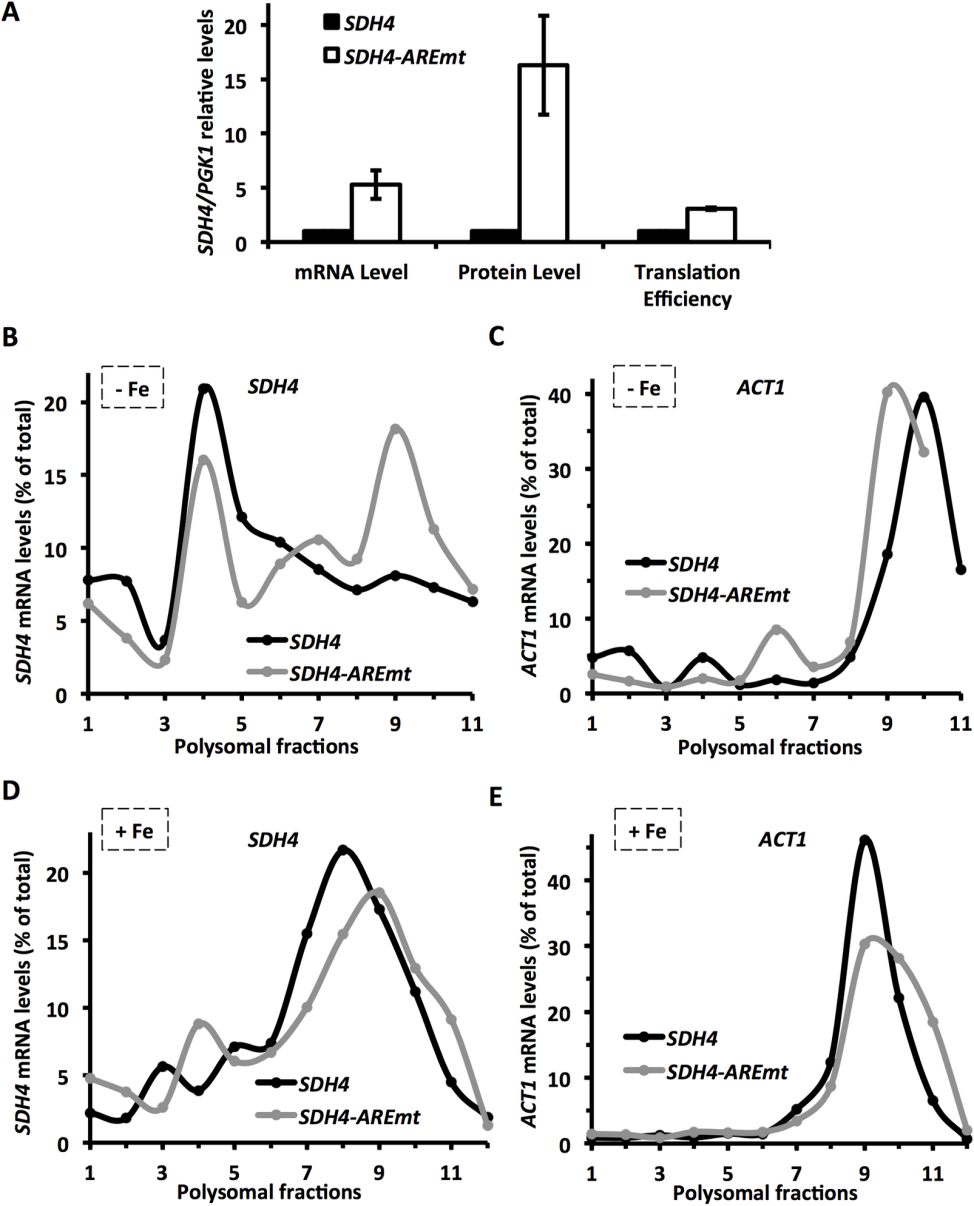
The *SDH4* mRNA AREs are required for its translational repression under iron-deficient conditions. **(A)** *sdh4Δ* cells transformed with plasmids pRS416-Flag_2_-SDH4 (*SDH4*) or pRS416-Flag_2_-SDH4-AREmt (*SDH4-AREmt*) were grown at 30°C for 7 hours in SC-Ura with 100 μM BPS. Flag_2_-Sdh4 and Pgk1 protein and mRNA levels were determined by Western blot and RT-qPCR, respectively. Translation efficiency was calculated as in Fig 1A. Mean values and standard deviations from two independent experiments are shown and referred to pRS416-Flag_2_-SDH4 (*SDH4*). **(B-E)** *sdh4Δ* mutant cells transformed with pRS416-SDH4 (*SDH4*) or pRS416-SDH4-AREmt (*SDH4-AREmt*) were grown under iron-deficient conditions as mentioned above (-Fe: B and C) or in SC-Ura (+Fe; D and E). Polysomal fractionation and analysis of *SDH4* (B, D) and *ACT1* (C, E) mRNA levels were carried out as described in Materials and Methods.

*SDH4* mRNA contains two AREs within its 3’-UTR. Since the degradation of *SDH4* mRNA under iron deficiency depends on these AREs [1], we wondered whether the negative regulation of *SDH4* translation observed under low iron conditions was also ARE-dependent. For this purpose yeast *sdh4Δ* cells expressing a plasmid–borne copy of *Flag*_*2*_*-SDH4* (*SDH4*) or *Flag*_*2*_*-SDH4* with four adenine to cytosine mutations introduced in its two AREs (*SDH4-AREmt*) were grown under low iron conditions for 7 h and translation efficiency was measured via the protein/mRNA ratio levels. In agreement with previous results [1], when the AREs within *SDH4* 3’-UTR were mutated, the *SDH4* mRNA levels displayed a 5-fold increase (Fig 2A). However, the Sdh4 protein levels rose ~16-fold compared to the wild-type *SDH4*, which resulted in a 3-fold increment in Sdh4 translation efficiency (Fig 2A). These results suggest that the AREs within the 3’-UTR of *SDH4* mRNA are involved in the negative regulation of the translation of *SDH4* under iron deficiency. This result was corroborated by the polysomal profile obtained for the *SDH4* and *SDH4-AREmt* mRNAs under iron-limited conditions. While wild-type *SDH4* mRNA was closely associated with the 80S peak, which is commonly interpreted as a translational inhibition occurring at the initiation step, the mutation of the AREs provoked a shift of *SDH4* mRNA toward the polysomal fractions, which is indicative of an amelioration of its translation (Fig 2B). Once again, the *ACT1* mRNA profile did not show any changes in its association with polysomes (Fig 2C).

We have previously established that *SDH4* mRNA is highly associated with polysomes under iron sufficiency. So we wondered whether AREs could affect the translation of *SDH4* mRNA under iron-repleted conditions. As shown in Fig 2D, the ARE mutations did not alter the *SDH4* mRNA profile, in a similar way to the control *ACT1* (Fig 2E). Therefore, we conclude that an ARE-mediated inhibition of *SDH4* translation takes place that is specific to iron-deficient conditions.

### Cth2 protein inhibits *SDH4* mRNA translation upon iron deficiency

**Fig 3:**
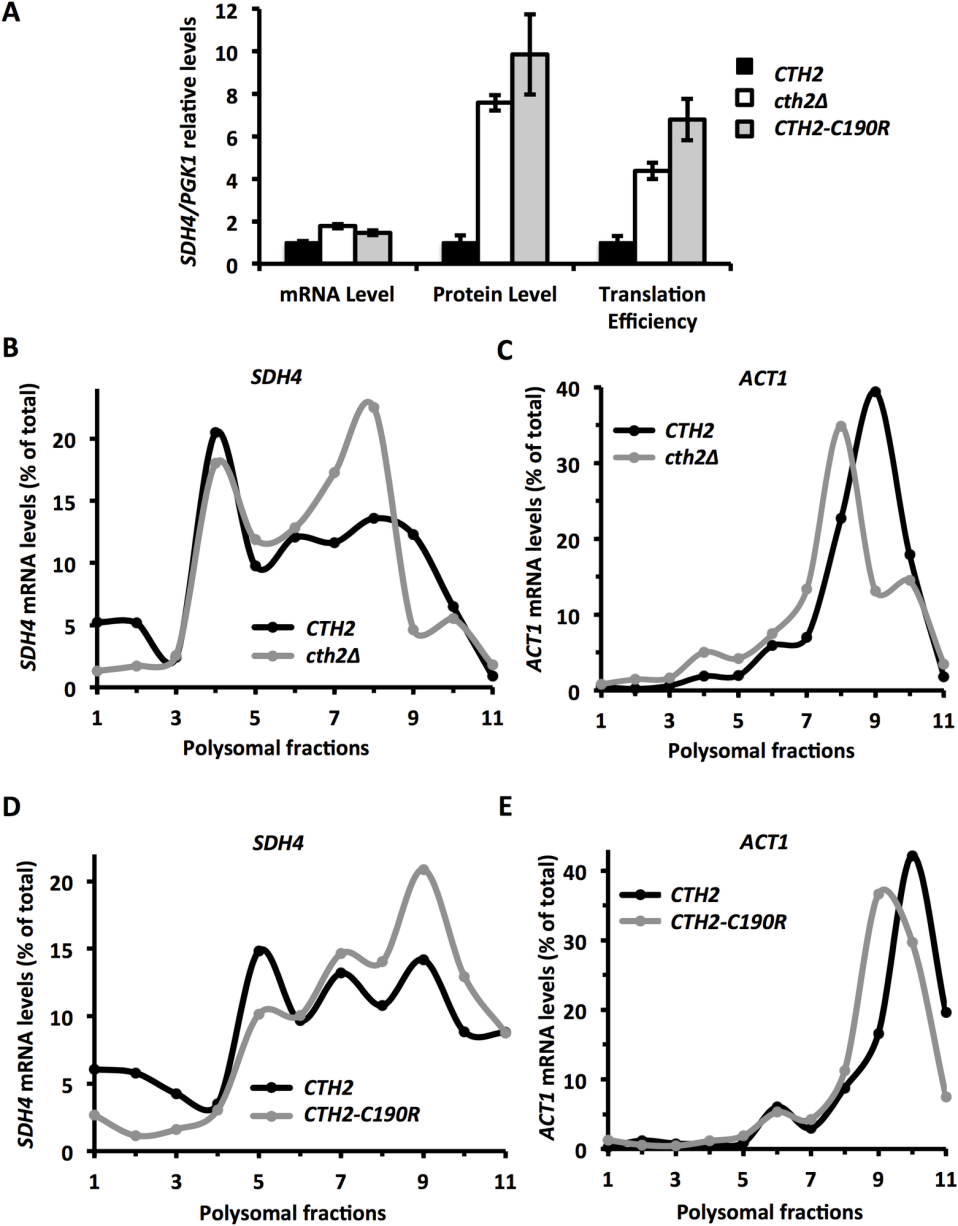
Cth2 represses *SDH4* mRNA translation in a Cth2-TZF-dependent manner when iron is scarce. **(A)** *cth1Δcth2Δ* mutant cells co-transformed with plasmids pRS416-Flag_2_-SDH4 and either pRS415-CTH2 *(CTH2)*, pRS415 (*cth2Δ*) or pRS415-CTH2-C190R *(CTH2-C190R)* were grown at 30°C for 7 h in SC-Ura-Leu supplemented with 100 μM BPS. Flag_2_-Sdh4 and Pgk1 protein/mRNA ratios were determined by Western blot/RT-qPCR, respectively, and translation efficiency was calculated as in Fig 1A. Mean values and standard deviation of three independent biological replicates are shown and referred to pRS415-CTH2 (*CTH2*). **(B and C)** Yeast *cth1Δcth2Δsdh4Δ* mutant cells co-transformed with plasmids pRS416-SDH4 and pRS415-CTH2 *(CTH2)* or pRS415 (*cth2Δ*) were cultivated as mentioned above and polysomal fractionation was carried out as described in Materials and Methods. The RNA in individual fractions was extracted and *SDH4* (B) and *ACT1* (C) mRNA levels were analyzed by RT-qPCR as described. **(D and E)** Yeast *cth1Δcth2Δsdh4Δ* mutant cells co-transformed with plasmids pRS416-SDH4 and pRS415-CTH2 *(CTH2)* or pRS415-CTH2-C190R *(CTH2-C190R)* were grown under iron-deficient conditions. Polysomal fractionation and RT-qPCR analysis were performed as above. Representative data from at least two independent experiments are shown.

In response to iron deficiency, the Cth2 protein binds the AREs in *SDH4* mRNA and promotes its degradation [1]. Since these AREs constitute the *cis* regulatory element involved in the translational regulation of *SDH4* mRNA by low iron, we decided to assess whether Cth2 was responsible for the iron-regulated repression of *SDH4* translation. To test this hypothesis, we analyzed *SDH4* translation efficiency via protein/mRNA levels in cells grown under iron-deficient conditions that coexpressed *Flag*_*2*_*-SDH4* either with *CTH2*, the empty vector, or with the TZF mutant *CTH2-C190R* that cannot bind mRNA. In order to exclude interferences from Cth1, which plays a secondary role in AMD under iron deficiency, all Cth2-related experiments described in this work were performed in a *cth1Δcth2Δ* background expressing different versions of Cth2 protein, as described [1,2,10,11]. As previously reported [1], in the absence of Cth2 (*cth2Δ*) or when Cth2 is not functional (*CTH2-C190R*), the *SDH4* mRNA levels increased ~1.5 to 1.8-fold (Fig 3A), whereas the Sdh4 protein levels showed a more marked increment (~8 to 10-fold). Consequently under iron-deficient conditions, *SDH4* translation efficiency augmented by 5 to 7-fold in the absence of a functional Cth2 protein. Then we determined the effect of Cth2 on the association of *SDH4* mRNA with the different polysomal fractions. As shown in Fig 3 (panels B and D), the association of *SDH4* mRNA with the heavier polysomal fractions was enhanced in both the absence of *CTH2* (*cth2Δ*) and the cells that expressed *CTH2-C190R*, grown under low iron conditions. Cth2 did not significantly alter the distribution profile of *ACT1* mRNA among the different polysomal fractions (Fig 3C and 3E). Taken together, these results demonstrate that *SDH4* translational repression under iron deficiency is mediated by the Cth2 protein through the binding of its TZFs to the AREs in the 3’-UTR of *SDH4* mRNA.

### *CTH2* mRNA translation is negatively regulated by the Cth2 protein

**Fig 4:**
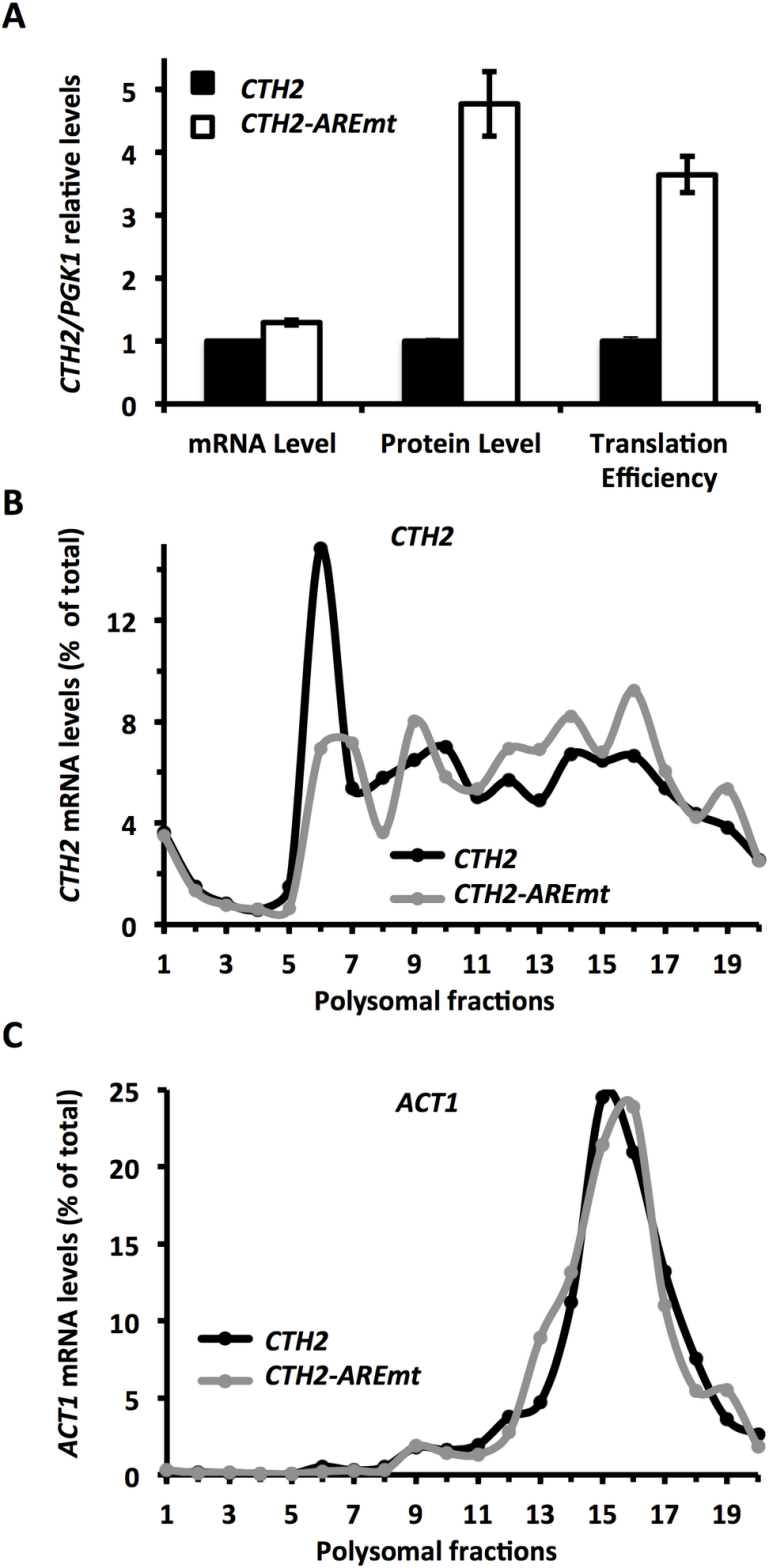
*CTH2* mRNA is translationally repressed by its ARE in low iron conditions. **(A)** *cth1Δcth2Δ* mutant cells transformed with pRS416-Flag_2_-CTH2 (*CTH2*) or pRS416-Flag_2_-CTH2-AREmt (*CTH2-AREmt*) were grown at 30°C for 7 hours in SC-Ura with 100 μM BPS (-Fe). Flag_2_-Cth2 and Pgk1 protein and mRNA levels were determined by Western blot and RT-qPCR, respectively. Flag_2_-*CTH2* translation efficiency was calculated as follows: (Flag_2_-Cth2 protein / Flag_2_-*CTH2* mRNA) / (Pgk1 protein / *PGK1* mRNA). Mean values and standard deviation from at least two independent experiments are shown and refer to pRS416-Flag_2_-CTH2 (*CTH2*). **(B and C)** *cth1Δcth2Δ* mutant cells transformed with pRS416-CTH2 (*CTH2*) or pRS416-CTH2-AREmt (*CTH2-AREmt*) were cultivated as mentioned above and polysomal fractionation was carried out as described in Materials and Methods. The RNA in individual fractions was extracted and percentages of *CTH2* (B) and *ACT1* mRNA (C) were analyzed by RT-qPCR as described in Materials and Methods. Representative data from at least two independent experiments are shown.

The Cth2 protein binds to the ARE within its own transcript to promote decay in an auto-regulated mechanism that limits its own expression [13]. This negative feedback loop allows a rapid response when iron supplementation occurs, due to the more rapid decline in Cth2 protein levels and the recovery of crucial iron-dependent processes such as respiration [13]. To address whether the AREs within *CTH2* mRNA were also involved in translational regulation, yeast cells that expressed either the *Flag*_*2*_*-CTH2* allele or its ARE mutant version *Flag*_*2*_-*CTH2-AREmt* were grown under iron-deficient conditions, and the translation efficiency of *CTH2* mRNA was analyzed. The *CTH2* mRNA levels increased 1.3-fold in the cells that expressed *CTH2-AREmt* compared to wild-type *CTH2*, while the protein levels increased 4.8-fold (Fig 4A). Thus *CTH2* mRNA translation efficiency was 3.6-fold better in the *CTH2-AREmt* cells than in the wild-type cells (Fig 4A). Once again, these results were confirmed by a slightly stronger presence of *CTH2* mRNA in heavy polysomal fractions and by a reduction in the monosomal fractions in those cells that expressed *CTH2-AREmt versus CTH2* (Fig 4B), while the *ACT1* mRNA profile was similar in both strains (Fig 4C). These results indicate that AREs act as a *cis-*regulatory element that is responsible for the translational repression of *CTH2* mRNA under iron deficiency.

**Fig 5:**
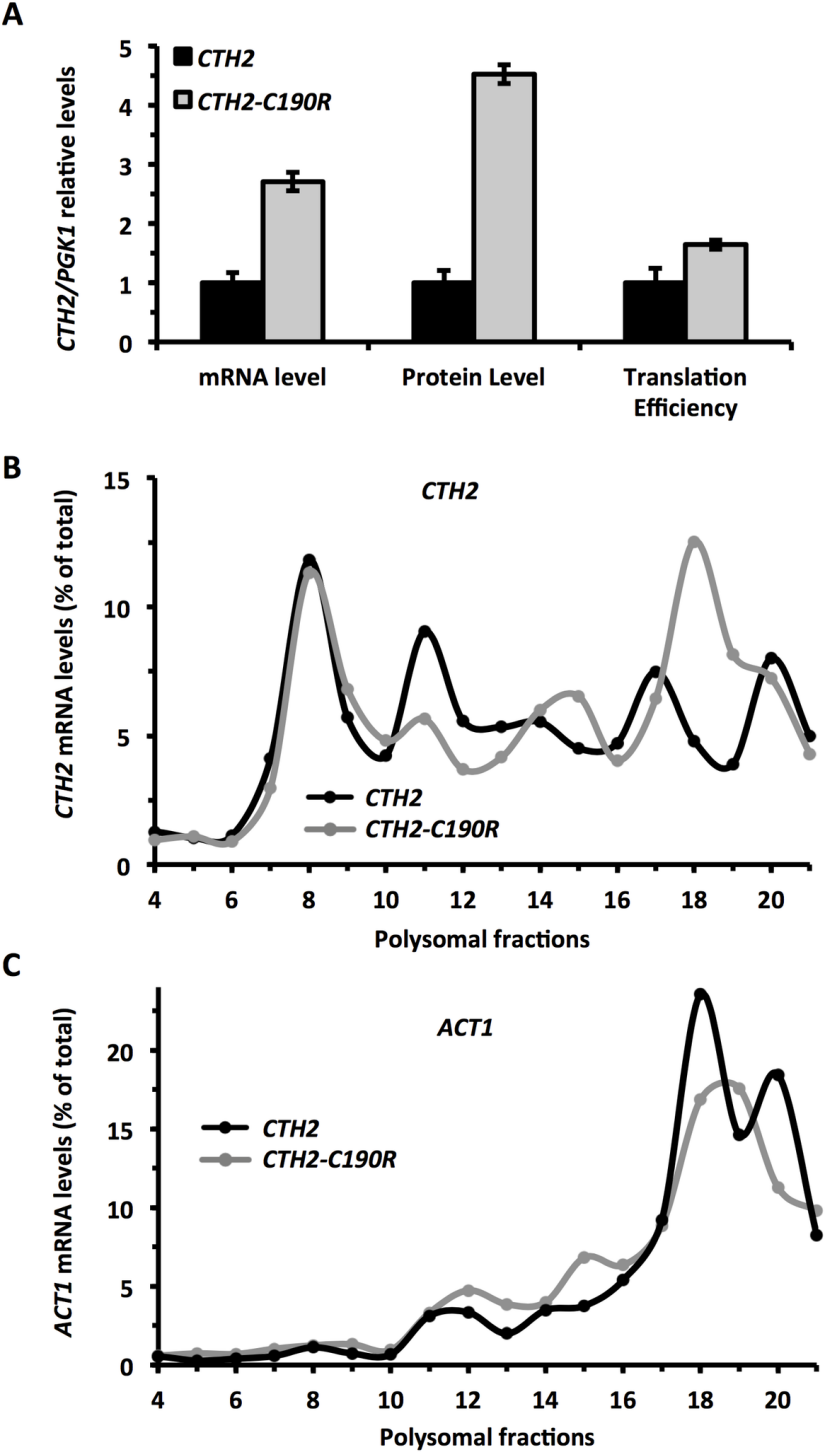
Cth2 represses its own mRNA translation in a Cth2-TZF-dependent manner under iron-limited conditions. **(A)** *cth1Δcth2Δ* mutant cells transformed with plasmids pRS416-Flag_2_-CTH2 (*CTH2*) or pRS416-Flag_2_-CTH2-C190R (*CTH2-C190R*) were grown at 30°C for 7 hours in SC-Ura with 100 μM BPS (-Fe). Mean values and standard deviation from four independent experiments of steady-state mRNA, proteins and *CTH2* translation efficiencies were determined and normalized as in Fig 4A. **(B and C)** *cth1Δcth2Δ* mutant cells transformed with pRS416-CTH2 (*CTH2*) or pRS416-CTH2-C190R (*CTH2-C190R*) were cultivated as mentioned above. Polysomal fractionation was carried out as described in Materials and Methods. The RNA of individual fractions was extracted and the percentages of *CTH2* **(B)** and *ACT1* mRNA **(C)** were analyzed by RT-qPCR as described in Materials and Methods. Representative data from at least two independent experiments are shown.

To assess whether the Cth2 protein was the *trans* factor that regulated *CTH2* mRNA expression at the translational level, we analyzed the translation efficiency of *CTH2* mRNA in yeast cells that expressed either the *Flag*_*2*_*-CTH2* or the *Flag*_*2*_*-CTH2-C190R* TZF mutant allele. In *CTH2-C190R* cells, the *CTH2* mRNA levels augmented 2.8-fold compared to the wild-type *CTH2* cells, whereas the protein levels increased 4.5-fold. If we consider that Cth2 protein stability was not altered by the C190R mutation (S1 Fig), we could conclude that *CTH2* translation efficiency was 1.6-fold higher when the Cth2 protein was unable to bind *CTH2* mRNA (Fig 5A). Consistently with these results, the *CTH2* mRNA profile after polyribosome fractioning showed a stronger association with the heavier polysome fractions in *CTH2-C190R* than in *CTH2* cells, while the *ACT1* mRNA profile was similar in both strains (Fig 5B and 5C). Taken together, these data strongly suggest that *CTH2* mRNA undergoes an auto-translational repression in iron deficiency, which is mediated by the specific binding of the Cth2 protein, via its TZF domain, to the AREs of *CTH2* transcript.

### Cth2 represses the translation of multiple ARE-containing mRNAs

**Fig 6:**
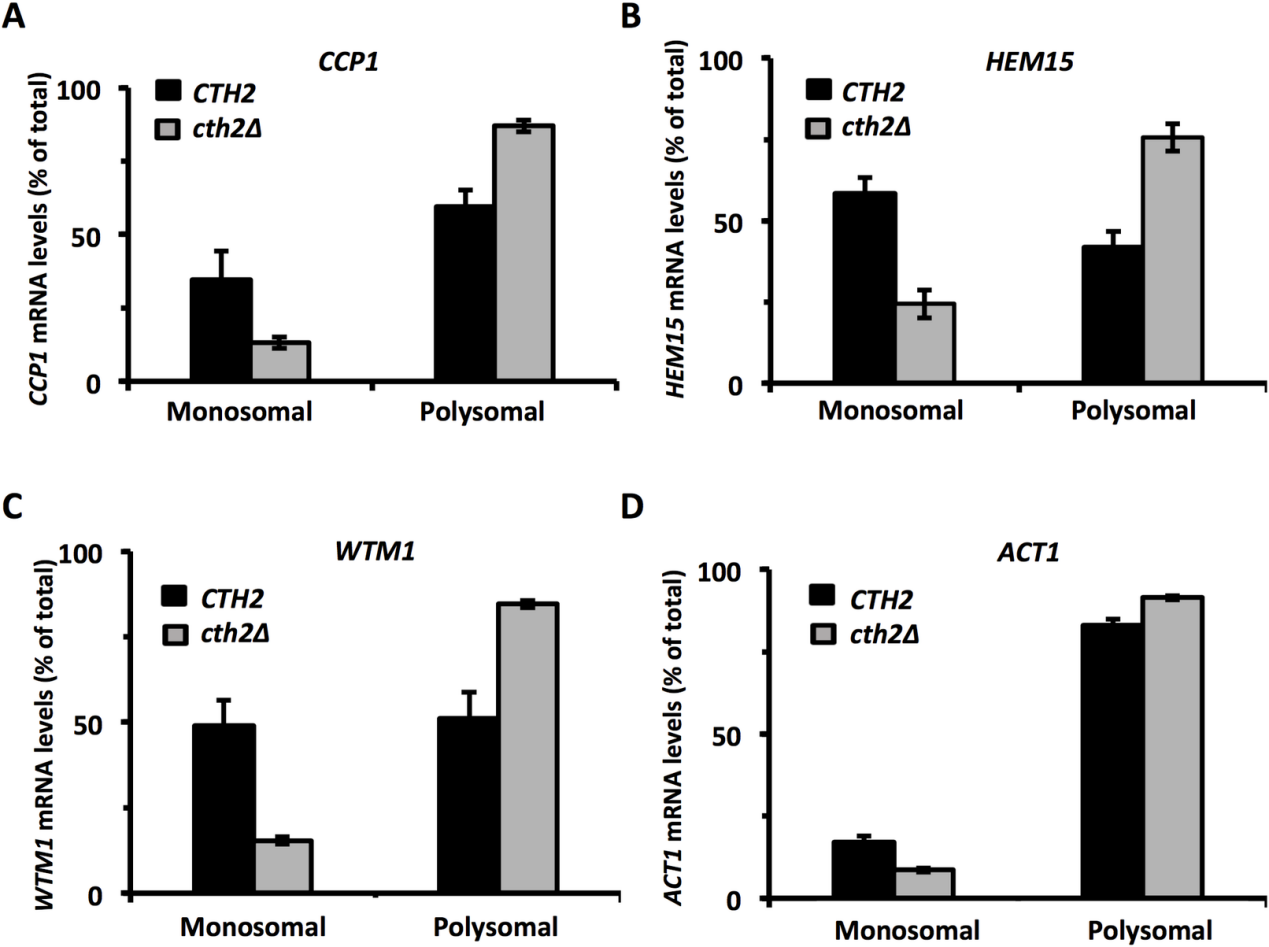
Cth2 represses the translation of other ARE-containing mRNAs under iron-limited conditions. *cth1Δcth2Δsdh4Δ* mutant strains co-transformed with pRS416-SDH4 and either pRS415-CTH2 *(CTH2)* or pRS415 (*cth2Δ*) plasmids were cultivated at 30°C for 7 h in SC-Ura-Leu with 100 μM BPS (-Fe). Polysomal fractionation was carried out as described in Materials and Methods. The percentages of endogenous *CCP1* **(A)**, *HEM15* **(B)**, *WTM1* **(C)** and *ACT1* **(D)** mRNAs from unified monosomal and polysomal fractions were determined by RT-qPCR as described in Materials and Methods. Mean values and standard deviations from three independent experiments are shown.

After demonstrating that *SDH4* and *CTH2* transcripts, both well-established targets of the Cth2 protein at the mRNA decay level, were also repressed at the translational level by Cth2-binding to their AREs, we aimed to study if this Cth2-dependent translational repression was a phenomenon that could extend to other Cth2 target mRNAs. For this purpose, we analyzed the polyribosomal profiles of additional Cth2 target-mRNAs in yeast cells that lacked (*cth2Δ*) or expressed *CTH2*. In order to more quantitatively address the association of these transcripts to ribosomes, the fractions that corresponded to monosomes and polysomes were pooled together prior to RNA extraction, and the mRNA association to each pool was determined. This analysis showed that the presence of *CCP1*, *HEM15* and *WTM1* mRNAs in the pooled polysomal *versus* monosomal fractions was significantly stronger in the absence of Cth2 (*cth2Δ*) compared to the wild-type *CTH2*-expressing cells (Fig 6A, 6B and 6C, respectively), while differences were minimal for the negative control *ACT1* (Fig 6D). In addition to this conjunct analysis, each fraction was separately extracted and independently analyzed, and the profiles obtained for these mRNAs qualitatively confirmed the increased association of *CCP1*, *HEM15* and *WTM1* mRNAs with the polysomal fractions in the cells that lacked *CTH2* compared to the *CTH2-*expressing cells (S2A, S2B and S2C Fig, respectively). These profiles were similar to those obtained for *SDH4* in this or previous experiments (S2E and S2F Fig and Fig 3B), while the non-iron related *ACT1* mRNA showed no Cth2-related differences in its association with polysomal fractions (S2D Fig). This analysis confirmed the results obtained with the pooled fractions, and supported that, in addition to *SDH4*, other previously described targets of Cth2 at the mRNA decay level, such as *CCP1*, *HEM15* and *WTM1*, were also regulated at the translational level, and showed a Cth2-dependent inhibition of translation under iron-deficient conditions.

### Cth2 NTD and CTD are implicated in *SDH4* translational repression, but only its NTD is responsible for mRNA decay

**Fig 7:**
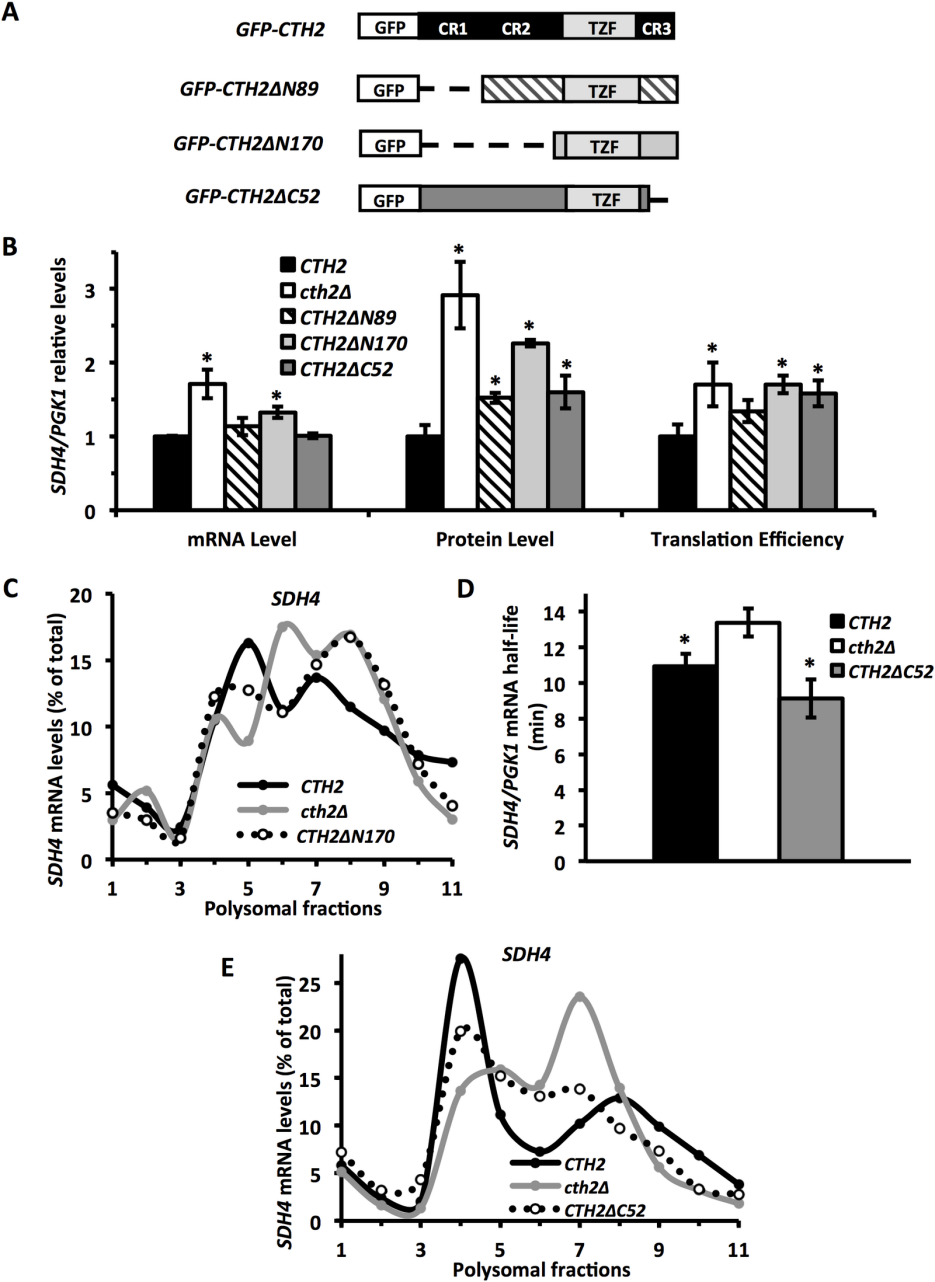
Both Cth2 NTD and CTD are necessary for *SDH4* mRNA translational repression, whereas only Cth2 NTD is implicated in *SDH4* mRNA decay upon iron starvation. **(A)** Schematic representation of the GFP-CTH2 fusion and truncations made at the NTD (*Δ*N) and CTD (*Δ*C) of Cth2 protein. **(B)** *cth1Δcth2Δ* mutant cells co-transformed with plasmids pRS415-Flag_2_-SDH4 and either pRS416-GFP-CTH2 (*CTH2*), pRS416 (*cth2Δ*), pRS416-GFP-CTH2*Δ*N89 (*CTH2ΔN89*), pRS416-GFP-CTH2*Δ*N170 (*CTH2ΔN170*) or pRS416-GFP-CTH2*Δ*C52 (*CTH2ΔC52*) were cultivated at 30°C for 7 h in SC-Ura-Leu with 100 μM BPS. Flag_2_-Sdh4 and Pgk1 protein and mRNA levels were determined by Western blot and RT-qPCR, respectively. Translation efficiency was calculated as in Fig 1A. Mean values and standard deviations from three independent experiments are shown and refer to pRS416-GFP-CTH2 (*CTH2*). An asterisk (*) indicates a significant *p*-value (≤ 0.03) compared with *CTH2*. **(C)** *cth1Δcth2Δsdh4Δ* mutant cells co-transformed with plasmids pRS415-SDH4 and either pRS416-GFP-CTH2 (*CTH2*), pRS416 (*cth2Δ*) or pRS416-GFP-CTH2*Δ*N170 (*CTH2ΔN170*) were cultivated at 30°C for 7 h in SC-Ura-Leu with 100 μM BPS. Polysomal fractionation was carried out as described in Materials and Methods. The RNA in individual fractions was extracted and the percentages of *SDH4* mRNA were analyzed by RT-qPCR as described in Materials and Methods. Representative data from at least two independent experiments are shown. **(D)** *cth1Δcth2Δsdh4Δ* mutant cells co-transformed with plasmids pRS415-GAL1-SDH4 and either pRS416-GFP-CTH2 (*CTH2*), pRS416 (*cth2Δ*) or pRS416-GFP-CTH2*Δ*C52 (*CTH2ΔC52*) were cultivated as described in Materials and Methods for the determination of *SDH4* mRNA half-life. Mean values and standard deviations from three independent experiments are shown. The asterisk (*) indicates a significant *p*-value (≤ 0.04) compared with c*th2Δ*. **(E)** *cth1Δcth2Δsdh4Δ* mutant cells co-transformed with plasmids pRS415-*SDH4* and either pRS416-GFP-CTH2 (*CTH2*), pRS416 (*cth2Δ*) or pRS416-GFP-CTH2*Δ*C52 (*CTH2ΔC52*) were cultivated and analyzed as in panel C. Representative data from at least two independent experiments are shown.

Previous studies have shown that the deletion of the 89 amino-terminal aminoacids of the Cth2 protein, which contains the conserved CR1 region, greatly impaired its ability to promote targeted mRNA degradation without affecting its shuttling between the nucleus and the cytoplasm [9,10]. This finding led us to hypothesize that the CR1 region was important for the Cth2 recruitment of components of the mRNA degradation machinery [9]. To decipher which Cth2 domains were important for its role in targeted translational repression, we tested the translation efficiency of *SDH4* mRNA under iron deprivation in the presence of different truncated versions of the Cth2 protein fused to GFP (Fig 7A: GFP-Cth2*Δ*N89, which lacks CR1; GFP-Cth2*Δ*N170, which lacks CR1 and CR2; and GFP-Cth2*Δ*C52, which lacks CR3). Similarly to previous experiments, we used the cells that expressed full-length *CTH2* (GFP-CTH2) or lacked *CTH2* as controls. We have previously shown that the amino-terminal fusion of GFP to Cth2 does not affect its function in mRNA decay and growth under iron-deficient conditions [10]. Furthermore, all the Cth2 truncated proteins used herein were able to shuttle between the nucleus and the cytoplasm ([10]; S3 Fig). In the absence of *CTH2* (*cth2Δ*), we observed a 1.7-fold increase in the *SDH4* mRNA levels compared to the cells that expressed *GFP-CTH2* (*CTH2*), while the Sdh4 protein levels rose 2.9-fold (Fig 7B). These data indicated that *SDH4* translation efficiency was enhanced by 1.7-fold in the *cth2Δ* cells (Fig 7B). When the cells that expressed *CTH2ΔN89* or *CTH2ΔN170* were analyzed, we observed an increase in *SDH4* mRNA and Sdh4 protein abundance, which did not reach the levels achieved by the cells that lacked *CTH2* (Fig 7B). It was noteworthy that *SDH4* translation efficiency slightly increased in *CTH2ΔN89* cells, and reached levels close to *cth2Δ* cells in *CTH2ΔN170* cells (Fig 7B). To address the contribution of Cth2 NTD to *SDH4* translational efficiency in more detail, we performed ribosomal profiles under low iron conditions in yeast cells that expressed *CTH2ΔN170* compared to the wild-type *CTH2* or *cth2Δ* cells (Fig 7C). We observed that the *SDH4* mRNA association with polysomes displayed by *CTH2-ΔN170* cells exhibited an intermediate profile between the profile of the *CTH2* and *cth2Δ* cells (Fig 7C). These results firmly suggest that both the conserved CR1 and CR2 NTDs of Cth2 contribute to Sdh4 translation inhibition.

We extended our study to Cth2 CTD, where an additional conserved CR3 region lies ([9]; Fig 7A). As previously reported, the steady-state *SDH4* mRNA levels were not altered when Cth2 CTD was deleted, which suggests that the CR3 region does not participate in mRNA decay ([9]; Fig 7B: *CTH2ΔC52*). To unequivocally determine whether Cth2 CTD contributed to *SDH4* mRNA stability, we analyzed the half-life of *SDH4* mRNA under iron-deficient conditions in cells that expressed wild-type *CTH2*, *CTH2ΔC52* or no *CTH2* (*cth2Δ*). The half-life of *SDH4* mRNA in the *CTH2ΔC52* mutant cells grown under iron deficiency conditions was similar to that observed in the wild-type *CTH2* cells, and lower than that of the *cth2Δ* cells (Fig 7D). Thus we can conclude that Cth2 CTD does not influence *SDH4* mRNA stability. However, protein quantification suggests that *SDH4* translation efficiency significantly augments in *CTH2ΔC52* cells (Fig 7B). To further address whether Cth2 CTD deletion led to improved *SDH4* translation, we performed ribosome profiles. We observed that the association of *SDH4* mRNA with the 80S monosomal peak weakened and shifted to heavier fractions in the yeast cells that expressed *CTH2ΔC52* (Fig 7E). Similarly to the *CTH2ΔN170* cells, the *CTH2ΔC52*-expressing strain displayed an intermediate distribution between the *CTH2* and *cth2Δ* cells (Fig 7E). Taken together, these results suggest that Cth2 NTD is important for both *SDH4* translational repression and decay, whereas CTD is involved in translation but not in mRNA decay.

### The Cth2 CTD is physiologically relevant under iron deficiency

**Fig 8:**
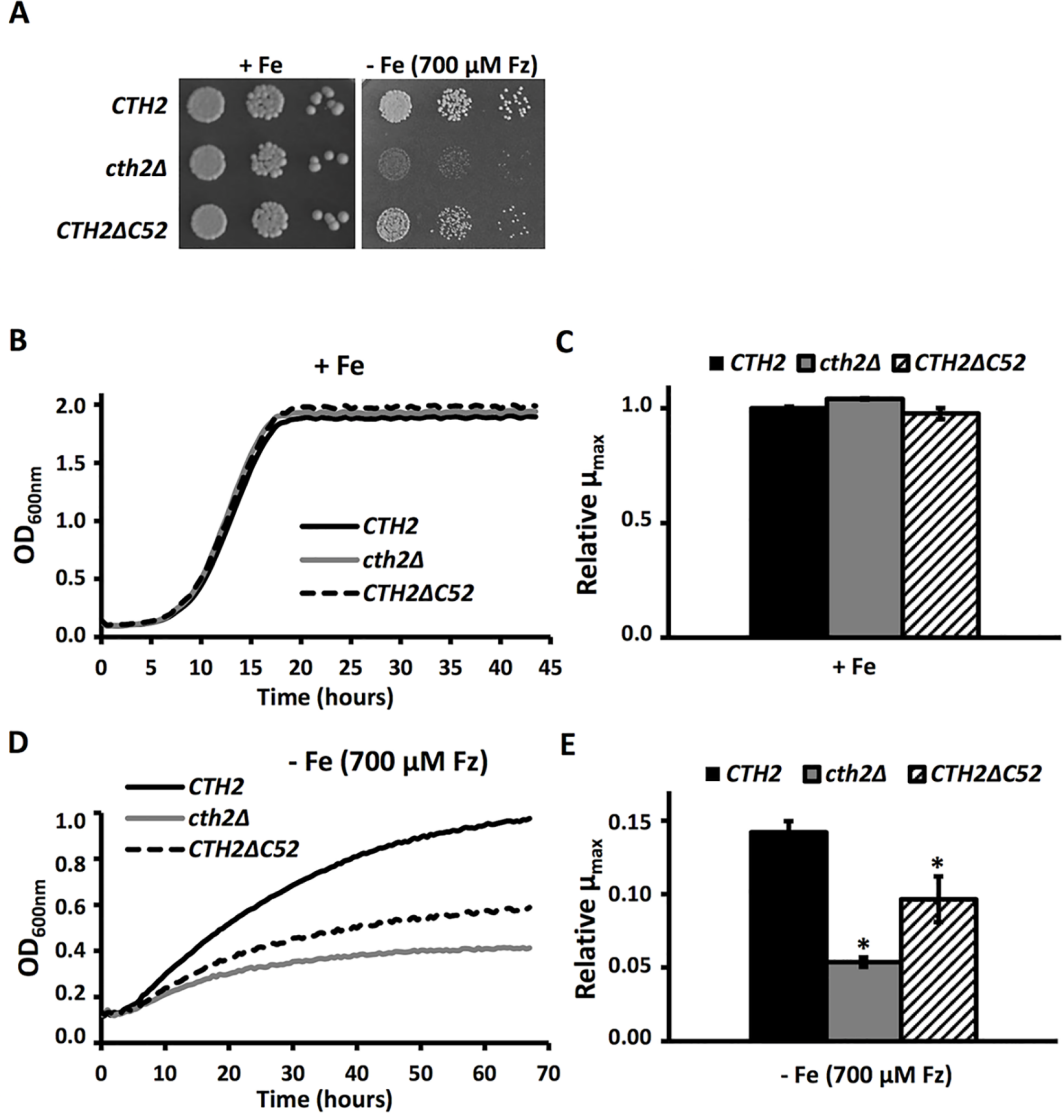
The Cth2 CTD mutant shows growth defects under iron-deficient conditions. **(A)** *cth1Δcth2Δ* mutant cells transformed with plasmids pRS416-GFP-CTH2 (*CTH2*), pRS416 (*cth2Δ*) or pRS416-GFP-CTH2*Δ*C52 (*CTH2ΔC52*) were grown to the exponential phase and spotted in 10-fold serial dilutions on SC-Ura and SC-Ura with 700 μM Ferrozine plates as described in Materials and Methods. (**B** and **D**) *cth1Δcth2Δ* mutant cells transformed with plasmids pRS416-GFP-CTH2 (*CTH2*), pRS416 (*cth2Δ*) or pRS416-GFP-CTH2*Δ*C52 (*CTH2ΔC52*) were inoculated at an OD_600nm_ of 0.1 in SC-Ura (**B**) and SC-Ura with 700 μM Ferrozine (**D**) and growth was determined by OD_600nm_ measurements as described in Materials and Methods. The growth curve values represented are the mean of three independent biological samples. (**C** and **E**) μ_max_ values for the growth curves represented in B (**C**) and D (**E**) were calculated as described in Materials and Methods. Mean values and standard deviations from at least three independent experiments are shown and referred to the value of *CTH2*-expressing cells in +Fe. The asterisk (*) indicates a significant *p*-value (≤ 0.02) compared with *CTH2* in -Fe.

Translational inhibition and mRNA decay are intimately coupled processes. However, we showed here that the Cth2 CTD is only implicated in the translational repression, but not the mRNA turnover, of the *SDH4* mRNA (Fig 7). To explore the contribution of the Cth2 CTD to the adaptation of yeast cells to iron depletion, we analyzed the growth of *CTH2ΔC52* cells as compared to cells lacking or expressing wild-type *CTH2*. As previously reported, *CTH2* expression did not influence yeast growth under iron-sufficient conditions ([1]; Fig 8A). Interestingly, the Cth2 CTD mutant showed a slight growth defect in a solid medium containing the Fe^2+^-specific chelator Ferrozine (Fig 8A). In order to further test this observation, we analyzed the growth of these strains in liquid medium, both in iron-sufficient and iron-deficient conditions (Fig 8B–8E). Again, no growth differences were observed under iron sufficiency (Fig 8B and 8C). However, when cultivated in iron-deprived conditions, the *CTH2ΔC52*-expressing cells displayed an important growth defect, which was reflected by both the maximum optical density at 600 nm (OD_600nm_) and the μ_max_ values achieved (Fig 8D and 8E). Taken together, these results support a physiologically relevant role for Cth2 CTD under iron deficiency, probably due to its defect in translational repression.

## Discussion

In response to iron deficiency, yeast cells activate the expression of *CTH2*, which encodes an RNA-binding protein that down-regulates multiple iron-dependent metabolic processes to optimize iron utilization [1]. When in the nucleus, Cth2 binds through its Cx_8_Cx_5_Cx_3_H-type TZFs to the AREs within the 3’-UTR of specific target mRNAs and promotes their decay by two described mechanisms [2]. In most cases, mRNA-bound Cth2 is exported to the cytoplasm where 5’ to 3’ degradation occurs [1,11]. Occasionally, Cth2 interferes with the polyadenylation site choice in its target transcripts and promotes the synthesis of extended mRNAs, which are rapidly degraded in the nucleus [9,10]. Several observations prompted us to propose that Cth2 could also regulate the translation efficiency of specific mRNAs. First, under iron-deficient conditions the mRNA levels of some Cth2 targets did not correlate with their corresponding protein amount [13]. Second, Cth2 genetically and physically interacted with Dhh1 RNA helicase, which is the yeast homolog of mammalian RCK/P54 translational repressor [11]. Third, in *xrn1Δ*, *dcp1Δ* and *dcp2Δ* mutant cells, Cth2 localized to processing-bodies, where transcripts temporarily incompetent for translation accumulate [33]. Here we have shown that Cth2 alters the fate of its target mRNAs by inhibiting their translation. This finding reinforces the importance of Cth2 in the repression of non-essential iron-consuming processes to facilitate the adaptation to iron scarcity.

By using protein/mRNA ratio measurements and by determining the mRNA association to different fractions of a polyribosome profile, we have concluded that *SDH4* mRNA translation greatly diminishes under iron-deficient conditions. We have observed that, under iron starvation, *SDH4* mRNA strongly associates to the monosome 80S peak and is scarce in the polysomal fractions (Fig 1). This profile is commonly interpreted as a translational repression that occurs in the initiation step, the most commonly regulated step in translation [34–38]. In principle, the shift of *SDH4* mRNA from polysomes to the 80S peak could be a consequence of the mild, but highly reproducible, global arrest of translation initiation that we have observed upon iron deficiency (Fig 1, 83% of polysomes in iron sufficiency *versus* 72% of polysomes in iron deficiency). However, this was not the case because the percentage of *SDH4* mRNA association to the heavy fractions of polysomes lowered from 79% in iron-sufficient conditions to 48% in iron deficiency, which represents a 3-fold greater decrease than the global profiles. On the other hand, *ACT1* mRNA changes its polysomal association from 93% to 82%, which represents a slight decrease that is quite correlative to the mild global change in polysome levels. These results strongly suggest that a specific translational regulation of *SDH4*, but not *ACT1*, mRNA occurs in response to iron deficiency.

In mammals, AREs participate in translation repression through the action of ARE-binding proteins such as TIA-1 and TIAR, and in translation activation via RNA-binding proteins of the ELAV family such as HuR [39–42]. More recently, Cth2 mammalian homolog TTP has been shown to repress the translation of ARE-containing transcripts with the help of the DEAD-box RNA helicase RCK/P54, or by entering in competition for ARE-binding with HuR [17,25]. Therefore, we decided to investigate whether the mechanisms that regulate *SDH4* mRNA translation, in an iron bioavailability-dependent manner, actually involved the AREs within its 3’-UTR. Our dual assay indicated that, under iron-deficient conditions, the mutations in the AREs of *SDH4* mRNA enhanced its translation efficiency (Fig 2). Interestingly, the ARE regulation of *SDH4* translation was not observed under iron-sufficient conditions, where *CTH2* expression is not detectable [1]; Fig 2). These observations strongly suggested that Cth2 could be the regulatory factor implicated in the *SDH4* translational regulation under iron scarcity. Once again the measurement of the protein/mRNA levels and polysome profiles indicated that *SDH4* translation improved in the absence of Cth2 or in the presence of a TZF-mutant Cth2 protein (Fig 3). Further analyses showed that the association with polysomes of other Cth2 mRNA targets, which participate in iron-dependent processes (*CCP1*, *HEM15*, *WTM1* and *CTH2* itself), increased when *CTH2* was not expressed (Figs 5 and 6). Altogether, these results suggest that Cth2 exerts both a general mRNA decay and translational repression of ARE-containing mRNAs that facilitate the adaptation to iron scarcity. Strikingly, the *SDH4* mRNA still shows a high association to the 80S monosome peak under iron-deficient conditions despite the lack of ARE (Fig 2B) or Cth1 and Cth2 proteins (Fig 3B and 3D). These results suggest that other currently unknown regulatory factors independent of Cth1 and Cth2, and functioning through cis elements different from the AREs, could be influencing the translation of the *SDH4* mRNA.

*CTH2* negative feedback regulation deserves special attention. We have previously reported that the Cth2 protein binds to an ARE within its own transcript and promotes its decay [13]. Now we know that *CTH2* auto-regulation also occurs at the translational repression level. This fine-tuned adjustment of Cth2 levels is especially relevant when yeast cells return to iron-sufficient conditions and a rapid shut-off of Cth2 protein becomes peremptory to reactivate Cth2-repressed processes, such as mitochondrial respiration, which are necessary for an optimal resumption of growth [13]. Yeast *CTH1* mRNA also contains functional AREs within its 3’-UTR that allow degradation via the Cth1 and Cth2 proteins [13]. In mammals, TTP also limits its own expression through an ARE-dependent feedback-regulatory loop at both the mRNA degradation and translational repression levels, which facilitates a rapid return to a resting state upon the removal of the activation signal [25,30,31]. Other TTP-family members, such as TIS11b and TIS11d, also possess AREs within the 3’-UTR of their respective mRNAs, and auto- and cross-regulate their expression in mouse embryonic stem cells [43]. It has been proposed that the interconnections between regulators that act on their own and on each other’s mRNAs would allow for an increased control and coordination of the expression of a broader set of mRNAs, which would thus permit faster metabolic adaptations in response to diverse environmental signals [44].

The Cth2-mediated regulation that we describe herein constitutes a novel example of translational regulation of iron metabolism in eukaryotes, which recalls the IRE/IRP system described in mammals. Although both regulatory networks act at the post-transcriptional level, the result of the IRE/IRP system depends on the mRNA region to which the corresponding IRP protein binds, with the 5’-UTR involved in translational inhibition and the 3’-UTR in mRNA stabilization (reviewed in [45–47]). In the case of the ARE/Cth2 system there is only one type of *cis* element situated in the 3’-UTR region of its target mRNAs [1]. As we show herein, Cth2 binding to AREs can mediate both translational repression and mRNA decay. Very little is currently known about the mechanisms by which Cth2 regulates both processes. Transcript half-life measurements have shown that the conserved CR1 within Cth2 NTD is important for targeted mRNA turnover, probably due to its implication in recruiting the components of the mRNA decay machinery [9]. Moreover, lack of Cth2 NTD leads to the formation of extended transcripts that are quickly degraded [9]. We decided to investigate which Cth2 domains were necessary for either mRNA turnover or translational inhibition. Our analysis showed that Cth2 NTD was important for both mRNA degradation and translation inhibition, while CTD was involved in translation regulation, but was dispensable for mRNA decay (Fig 7). Importantly, the results obtained with the Cth2 CTD mutant suggest that the role of Cth2 in translational repression is physiologically relevant, which greatly supports the intrinsic importance of Cth2 function as a translational regulator.

Transcript translation and degradation are intimately related processes, with mRNA decay generally occurring as a consequence of translational repression [48,49]. For instance, the decapping activators Dhh1 slows down the movement of ribosomes on mRNAs during the elongation phase of translation, prior to the activation of decapping and independently of it [50,51]. However, there are also examples where both processes have been separated. For example, a distinct role of some ARE-binding proteins in translation inhibition, but not in mRNA decay, has been established [52]. In the case of Cth2, we currently do not know which process takes place first, activation of decay or translational repression. Our results indicate that distinctive partner/s may interact with Cth2 CTD to regulate translation, while other common factors would possibly be implicated in both decay and translation through interactions with Cth2 NTD. Further work is necessary to identity Cth2 trans-acting factors, to distinguish their general regulatory function from its Cth2-dependent role, and to characterize the initial events that lead to Cth2 repression of gene expression.

## Materials and methods

### Yeast strains, plasmids and growth conditions

BY4741 wild-type (*MATa his3Δ1 leu2Δ0 met15Δ0 ura3Δ0*) and *sdh4Δ* mutant (BY4741 *sdh4*::*KanMX4*) strains were obtained from Research Genetics. *cth1Δcth2Δ* (BY4741 *cth1*::*KanMX4 cth2*::*HisMX6*) and *cth1Δcth2Δsdh4Δ* (BY4741 *cth1*::*KanMX4 cth2*::*HisMX6 sdh4*::*hphB*) mutant strains have been previously described [1]. For the RNA, protein and polyribosome profile analyses, yeast cell precultures transformed with specific plasmids (listed in S2 Table) were incubated overnight at 30°C in synthetic complete medium lacking specific requirements (SC minus). They were then reinoculated at OD_600nm_ = 0.2–0.4 and incubated 7 h in SC minus supplemented with 10 μM ferrous ammonium sulfate or FAS (iron-sufficient conditions: +Fe) or SC minus supplemented with 100 μM of the Fe^2+^-specific chelator bathophenanthroline disulfonate or BPS (iron-deficient conditions: -Fe) to exponential phase. For fluorescence microscopy, yeast cells were cultivated in SC-Ura supplemented with 100 μM BPS to early exponential phase. For growth assays in liquid media, yeast cells were inoculated in 96-well plates at an OD_600nm_ of 0.1 in 260 μL of liquid SC-Ura medium without (+Fe) or with 700 μM Ferrozine (-Fe), and the OD_600nm_ was determined in a Spectrostar Nano absorbance microplate reader (BMGLabtech) every 30 min for 2–3 days at 28°C. μ_max_ is the maximum specific growth rate (h^−1^) and was calculated from each condition by directly fitting OD_600nm_ measurements *versus* time to the reparameterized Gompertz equation [53], ln(OD_t_/OD_0_) = D * exp {-exp[((μ_max_*e)/D)*(λ - t) + 1]}, where OD_0_ is the initial OD and OD_t_ is the OD at time t; D = ln(OD_∞_/OD_0_) is the OD value reached with OD_∞_ as the asymptotic maximum, μ_max_ is the maximum specific growth rate (h^−1^), and λ is the lag phase period (h). Growth assays in solid media (1.5% agar) were performed as previously described [54]. Cells were cultivated to exponential phase, diluted to an OD_600nm_ of 0.1 and then spotted directly and after 1:10 and 1:100 dilutions in SC-Ura (+Fe) or SC-Ura with 700 μM Ferrozine (-Fe). Solid media were incubated at 30°C and then photographed.

### RNA analyses

Total yeast RNA isolation, reverse transcription and quantitative real-time PCR (RT-qPCR) were performed as previously described [55]. An oligo-dT primer was used for the reverse transcription, and specific primer pairs were used for the RT-qPCR (listed in S1 Table). The data and error bars represent the relative average and standard deviations of at least two independent biological samples.

For mRNA half-life determination, yeast cells were grown overnight in SC-Ura-Leu-raffinose (2% [w/v] raffinose, no glucose) and were reinoculated in SC-Ura-Leu-galactose (2% [w/v] galactose, no glucose) supplemented with 100 μM BPS at 30°C until exponential growth phase (OD_600nm_ of 0.4–0.6, approximately 4 h). Then, glucose was added to a final concentration of 4% [w/v] to inhibit the transcription of the *P*_*GAL1*_*-SDH4* fusions. After glucose addition, aliquots were isolated at successive times (0, 5, 10 and 15 min), total RNA was extracted and cDNA was obtained as described above with one modification, the use of random primers instead of oligo-dT. The cDNA was then analyzed by RT-qPCR using specific primer pairs (listed in S1 Table). *SDH4* mRNA levels were normalized to *PGK1* mRNA levels. The mRNA half-life was determined from three independent experiments. Tailed t-student tests were applied to evaluate statistical significance.

### Protein analyses

Total protein extracts were obtained by using the alkali method [56]. Equal amounts of protein were resolved in 10% SDS-PAGE gels and transferred to nitrocellulose membranes. Ponceau staining was used to assess protein transfer. Flag_2_-Sdh4 and Flag_2_-Cth2 were detected using a horseradish peroxidase (HRP)-conjugated anti-Flag antibody (A8592; Sigma). Anti-Pgk1 primary antibody (22C5D8; Invitrogen) and HRP-labeled secondary anti-mouse antibody (GE Healthcare Life Sciences) were used to determine the Pgk1 protein levels as loading control. ECL Select Western blotting detection kit was used (GE Healthcare Life Sciences). Immunoblot images were obtained in an ImageQuant LAS 4000 mini Biomolecular Imager (GE Healthcare Life Sciences) and specific signals were quantified with ImageQuant TL analysis software (GE Healthcare Life Sciences). Flag_2_-*SDH4* translation efficiency was calculated as: (Flag_2_-Sdh4 protein / Flag_2_-*SDH4* mRNA) / (Pgk1 protein / *PGK1* mRNA) and Flag_2_-*CTH2* translation efficiency was calculated as: (Flag_2_-Cth2 protein / Flag_2_-*CTH2* mRNA) / (Pgk1 protein / *PGK1* mRNA).

For protein half-life determination purposes, cells were grown exponentially in SC-Ura supplemented with 10 μM FAS and in SC-Ura supplemented with 100 μM of BPS for 7 h as described above. Protein translation was stopped by adding cycloheximide (CHX) to a final 50 μg/mL concentration. The Flag_2_-Sdh4, Flag_2_-Cth2 and Flag_2_-Cth2-C190R protein levels were determined at successive times (0, 5, 15, 30 and 60 min) after adding CHX. A nonspecific anti-FLAG band was used as loading control in Flag_2_-Sdh4 half-life determination.

### Polyribosome profile analysis

Cells were grown to the exponential phase in SC minus the specific requirements and were supplemented with 100 μM of BPS for 7 h. Preparation of cells and polysome gradients were performed as described by Garre *et al*. [57], with some modifications. A culture volume that corresponded to an OD_600nm_ of 60 was chilled for 5 min on ice in the presence of 0.1 mg/mL CHX. Cells were harvested by centrifugation at 6000 × g for 4 min at 4°C and washed twice with 1 mL of lysis buffer (20 mM Tris-HCl, pH 8, 140 mM KCl, 5 mM MgCl2, 0.5 mM dithiothreitol, 1% Triton X-100, 0.1 mg/mL CHX, and 0.5 mg/mL heparin). Cells were resuspended in 700 μL of lysis buffer, a 0.5-mL volume of glass beads was added, and cells were disrupted by vortexing 8 times for 30 s with 30 s of incubation on ice in between. Lysates were cleared by centrifugation at 5000 rpm for 5 min at 4°C, after which the supernatant was recovered and centrifuged at 8000 rpm for 5 min at 4°C. Finally, glycerol was added to the supernatant at a final concentration of 5% and extracts were frozen in liquid nitrogen and stored at -70°C. Samples of 8.5 absorbance at 260 nm (A_260nm_) units were loaded onto 5–50% sucrose gradients and were separated by ultracentrifugation for 2 h 40 min at 35000 rpm in a Beckman SW41 rotor at 4°C. Gradients were then fractionated by isotonic pumping of 60% sucrose from the bottom and either eleven 1 mL-samples or twenty-two 0.5 mL-samples were recovered. The polysomal profiles were monitored by online UV detection at 260 nm (Density Gradient Fractionation System; Teledyne Isco, Lincoln, NE). For the RNA analyses of the polysomal fractions, 8 μL of mixed *lys* and *spo* mRNAs at 3 ng/ μL from *Bacillus subtilis* were added to 200 μL of each fraction before extraction. RNA was extracted using SpeedTools Total RNA Extraction kit (Biotools B&M Labs) with the rDNAse treatment after the RNA elution step. For the unified monosomal and polysomal fractions, equal volumes of each fraction were combined in a final 200 μL volume and RNA was extracted with the same kit. Specific mRNAs were analyzed by RT-qPCR using specific primer pairs (listed in S1 Table) and represented as a percentage of total. All the values were normalized with spiked-in mRNA levels of *B*. *subtilis lys* and *spo*.

### Fluorescence microscopy

Yeast cells transformed with GFP-containing plasmids were visualized in an Axioskop 2 fluorescence microscope (Zeiss). Images were captured with a SPOT camera (Diagnostic Instruments).

## Supporting information

S1 Fig Protein half-life of Cth2 and Cth2-C190R proteins in iron-deficient conditions. Yeast *cth1Δcth2Δ* cells transformed with pRS416-Flag_2_-CTH2 or pRS416-Flag_2_-CTH2-C190R plasmids were cultivated in SC-Ura with 100 μM BPS for 6 h to exponential phase. Then, 50 μg/mL cycloheximide (CHX) was added to stop translation, and aliquots were isolated at the indicated times. Total proteins were extracted, and Cth2 protein levels were determined by immunoblotting with anti-Flag antibody. Equal amounts of total proteins were loaded in each lane. Cth2 and Cth2-C190R protein levels were determined and the mean values of Cth2 and Cth2-C190R protein half-life (t_1/2_) from two independent experiments were calculated. A representative experiment is shown. The asterisk (*) indicates a non-specific band.
(TIFF)

S2 Fig Cth2 represses the translation of *SDH4* mRNA in iron deprivation. *cth1Δcth2Δsdh4Δ* mutant cells were co-transformed and cultivated as in Fig 6. The RNA in individual fractions was extracted and the percentages of endogenous *CCP1* **(A)**, *HEM15* **(B)**, *WTM1* **(C)**, *ACT1* **(D) and *SDH4* (E)** mRNAs were analyzed by RT-qPCR as described in Materials and Methods. Representative data from at least two independent experiments are shown. The percentages of endogenous *SDH4* **(F)** mRNAs from unified monosomal and polysomal fractions were determined by RT-qPCR as described in Materials and Methods. Mean values and standard deviations from three independent experiments are shown.
(TIFF)

S3 Fig Cth2 truncated proteins localize to both the nucleus and the cytoplasm. Yeast *cth1Δcth2Δ* cells transformed with pRS416-GFP-CTH2 (*GFP*-*CTH2*), pRS416-GFP-CTH2*Δ*N89 (*GFP*-*CTH2ΔN89*), pRS416-GFP-CTH2*Δ*N170 (GFP-*CTH2ΔN170*) or pRS416-GFP-CTH2*Δ*C52 (*GFP*-*CTH2ΔC52*) plasmids were cultivated at 30°C in SC-Ura with 100 μM BPS for 6 h to reach early exponential phase, and cells were analyzed with the fluorescence microscope. Representative images corresponding to green fluorescence (GFP) and differential interference microscopy (DIC) are shown.
(TIFF)

S1 Table Primer pairs used in RT-qPCR in this study. (DOCX)

S2 Table Plasmids used in this study. (DOCX)
