# Supplementary figures and images for: Yeast Cth2 protein represses the translation of ARE-containing mRNAs in response to iron deficiency
Source: PLoS Genet. 2018 Jun 18;14(6):e1007476. doi: 10.1371/journal.pgen.1007476 (PMC6023232; doi:10.1371/journal.pgen.1007476)

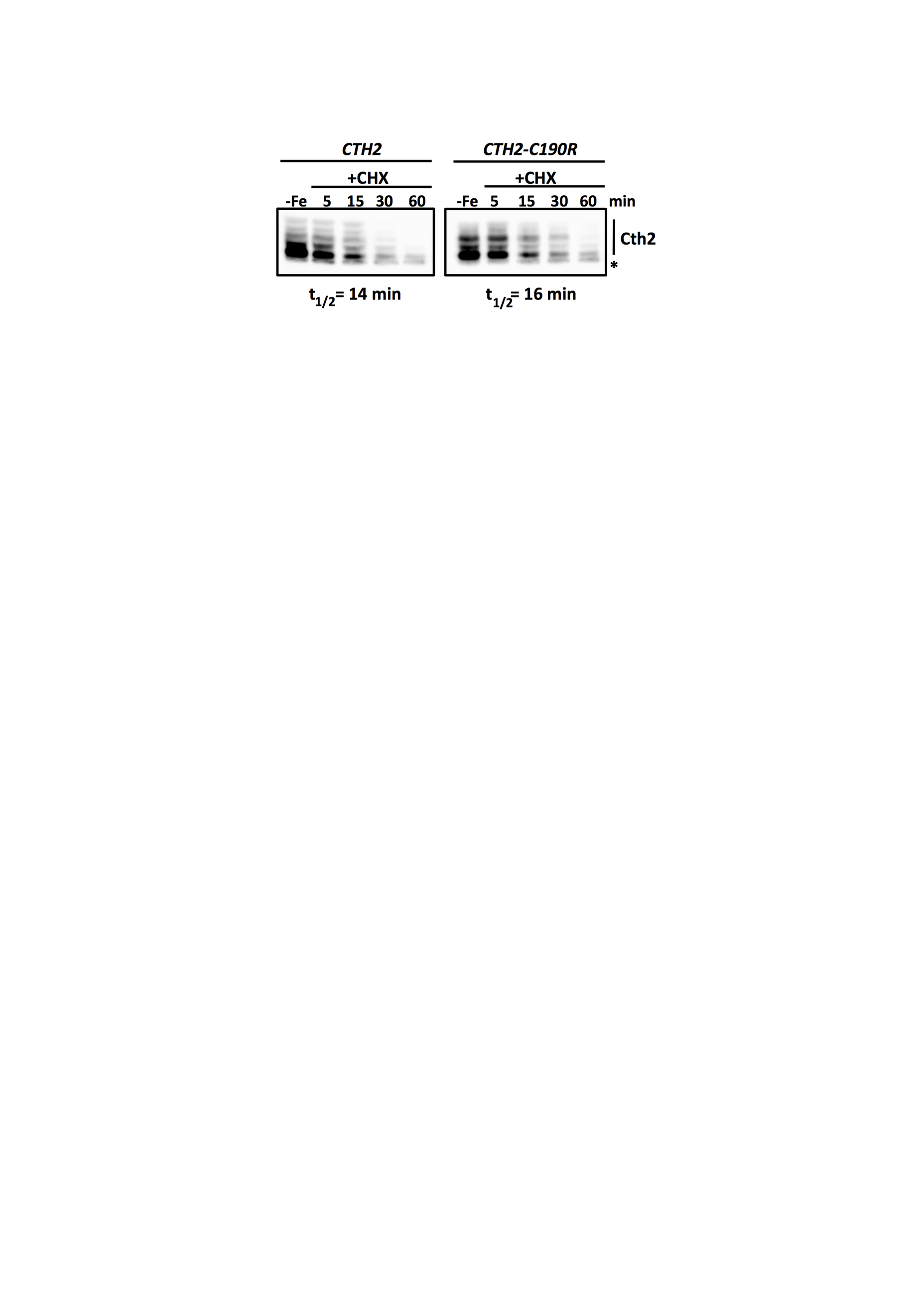

Supplement: S1 Fig — Yeast cth1Δcth2Δ cells transformed with pRS416-Flag2-CTH2 or pRS416-Flag2-CTH2-C190R plasmids were cultivated in SC-Ura with 100 μM BPS for 6 h to exponential phase. Then, 50 μg/mL cycloheximide (CHX) was added to stop translation, and aliquots were isolated at the indicated times. Total proteins were extracted, and Cth2 protein levels were determined by immunoblotting with anti-Flag antibody. Equal amounts of total proteins were loaded in each lane. Cth2 and Cth2-C190R protein levels were determined and the mean values of Cth2 and Cth2-C190R protein half-life (t1/2) from two independent experiments were calculated. A representative experiment is shown. The asterisk (*) indicates a non-specific band. (TIFF) [file pgen.1007476.s001.tiff]

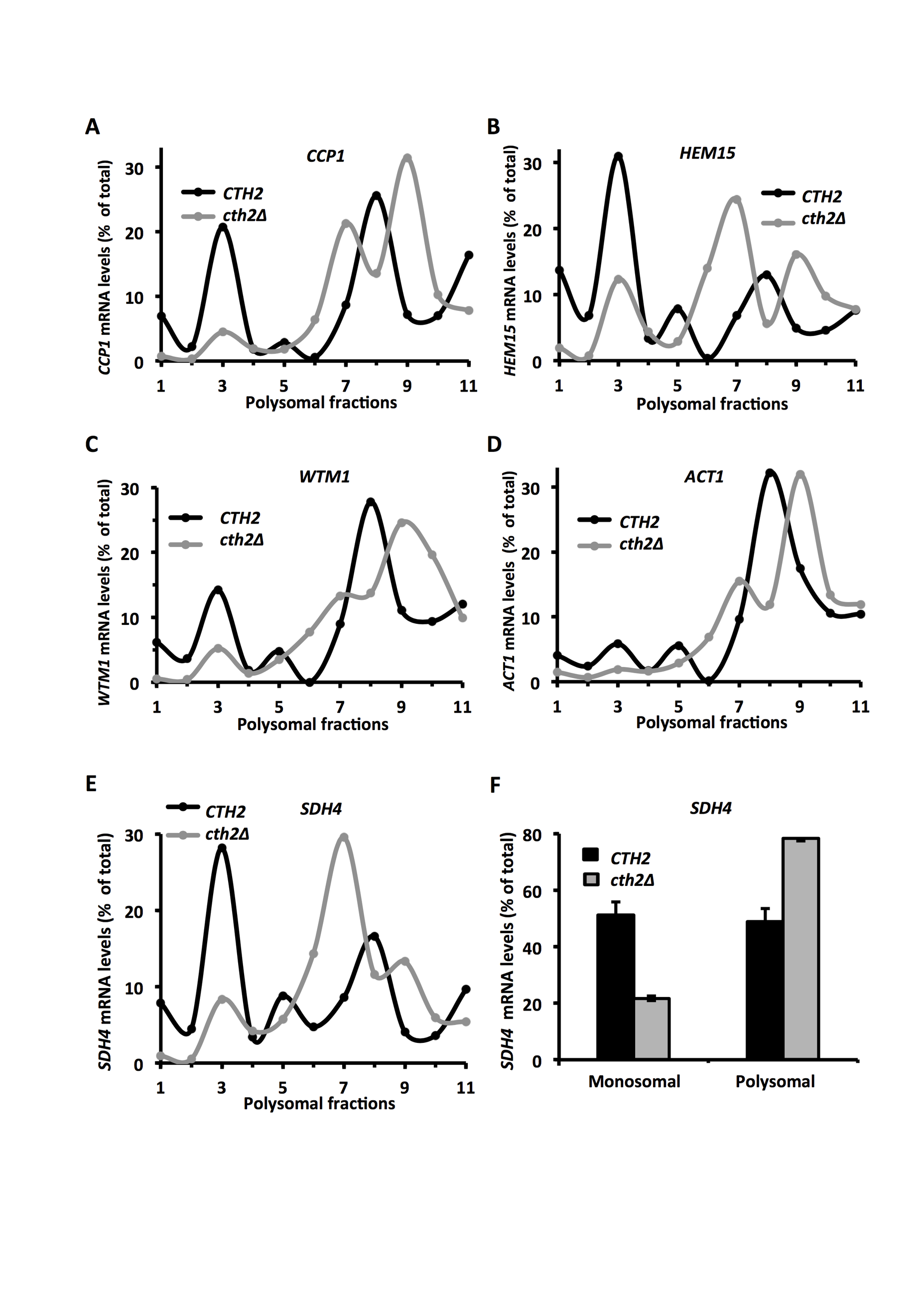

Supplement: S2 Fig — cth1Δcth2Δsdh4Δ mutant cells were co-transformed and cultivated as in Fig 6. The RNA in individual fractions was extracted and the percentages of endogenous CCP1 (A), HEM15 (B), WTM1 (C), ACT1 (D) and SDH4 (E) mRNAs were analyzed by RT-qPCR as described in Materials and Methods. Representative data from at least two independent experiments are shown. The percentages of endogenous SDH4 (F) mRNAs from unified monosomal and polysomal fractions were determined by RT-qPCR as described in Materials and Methods. Mean values and standard deviations from three independent experiments are shown. (TIFF) [file pgen.1007476.s002.tiff]

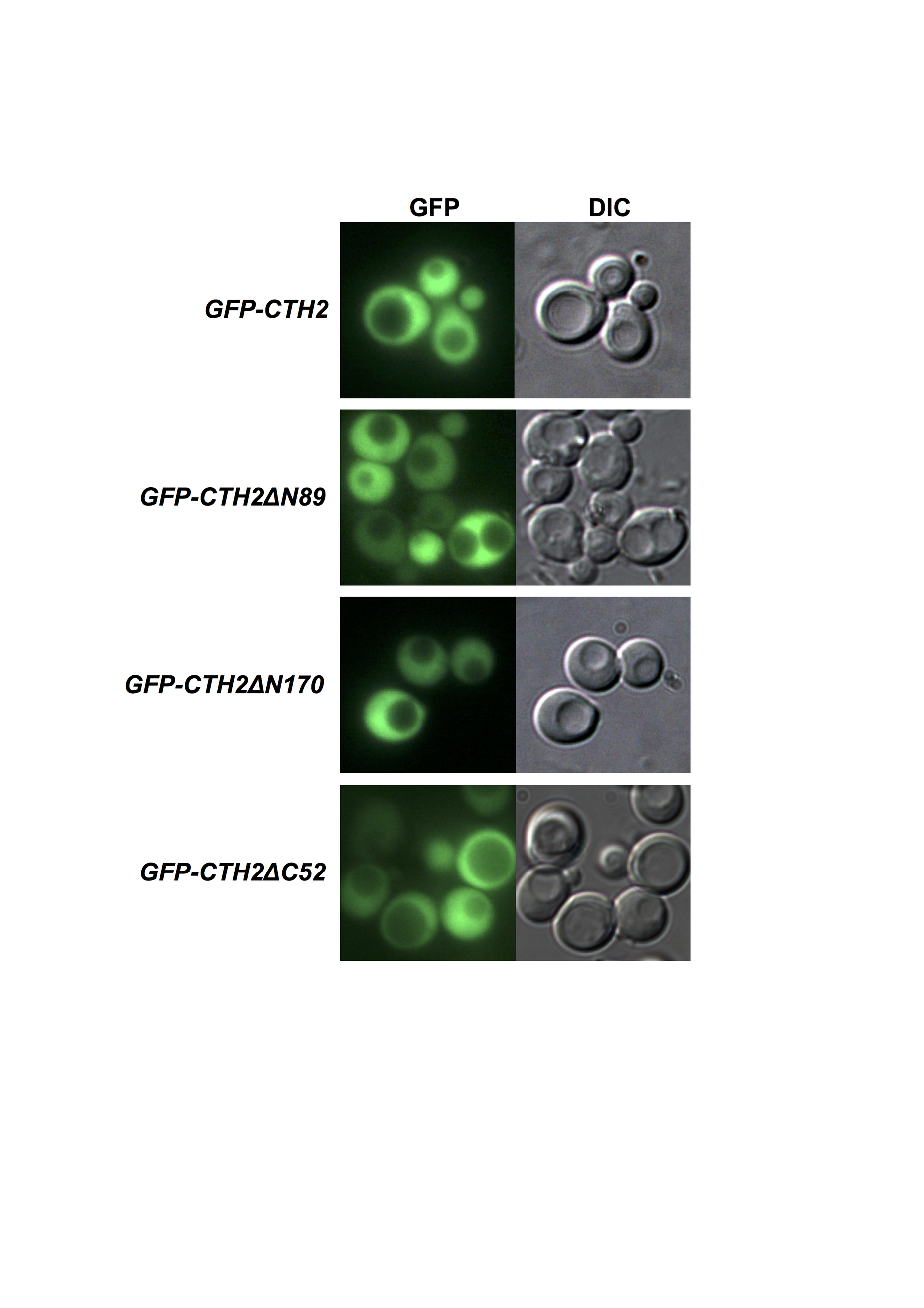

Supplement: S3 Fig — Yeast cth1Δcth2Δ cells transformed with pRS416-GFP-CTH2 (GFP-CTH2), pRS416-GFP-CTH2ΔN89 (GFP-CTH2ΔN89), pRS416-GFP-CTH2ΔN170 (GFP-CTH2ΔN170) or pRS416-GFP-CTH2ΔC52 (GFP-CTH2ΔC52) plasmids were cultivated at 30°C in SC-Ura with 100 μM BPS for 6 h to reach early exponential phase, and cells were analyzed with the fluorescence microscope. Representative images corresponding to green fluorescence (GFP) and differential interference microscopy (DIC) are shown. (TIFF) [file pgen.1007476.s003.tiff]
