# Supplementary material for: Yeast Cth2 protein represses the translation of ARE-containing mRNAs in response to iron deficiency
Source: PLoS Genet. 2018 Jun 18;14(6):e1007476. doi: 10.1371/journal.pgen.1007476 (PMC6023232; doi:10.1371/journal.pgen.1007476)
Supplement: S1 Table — (DOCX) [file pgen.1007476.s004.docx]

| **S1 Table. Primer pairs used in RT-qPCR in this study** | |
| --- | --- |
|  |  |
|  |  |
| Primer | Sequence |
| *SDH4*-F | 5'-GCACTCCCAATGATGCCTAC-3' |
| *SDH4*-R | 5'-AATGGAACGACGGACAAGG-3' |
| Flag_2_-*SDH4*-F | 5'-TTGATCTTTCCTACGCTTTCG-3' |
| Flag_2_-*SDH4*-R | 5'-TCGTCCTTGTAGTCGCCTTT-3' |
| *CTH2*-F | 5'-GCAGTTTCATTCTCTCCAC-3' |
| *CTH2*-R | 5'-TAGGTGCCGTGCTATTCAGG-3' |
| *PGK1*-F | 5'-AAGCGTGTCTTCATCAGAGTTG |
| *PGK1*-R | 5'-CGTATCTTGGGTGGTGTTCC-3' |
| *ACT1*-F | 5'-TCGTTCCAATTTACGCTGGTT-3' |
| *ACT1*-R | 5'-CGGCCAAATCGATTCTCAA-3' |
| *CCP1*-F | 5'-ACTCGCAATCCCAAAAGAGA |
| *CCP1*-R | 5'-CGGTGTAGTGGAAGCCAAAG-3' |
| *HEM15*-F | 5'-CCAAAGTTGATGGCCTAATG-3' |
| *HEM15*-R | 5'-TATTCCGATTCCCCAATGAC-3' |
| *WTM1*-F | 5'-TCCTGACGATACCATTGCTC-3' |
| *WTM1*-R | 5'-TCTTCTGCTTCCACCCTTGT-3' |
|  |  |
