# Supplementary material for: Yeast Cth2 protein represses the translation of ARE-containing mRNAs in response to iron deficiency
Source: PLoS Genet. 2018 Jun 18;14(6):e1007476. doi: 10.1371/journal.pgen.1007476 (PMC6023232; doi:10.1371/journal.pgen.1007476)
Supplement: S2 Table — (DOCX) [file pgen.1007476.s005.docx]

| **S2 Table. Plasmids used in this study** | |  |
| --- | --- | --- |
|  |  |  |
| Plasmid | Description | Source/Reference |
| pRS415 | CEN *LEU2* | [58] |
| pSP949 | pRS415-Flag_2_-*SDH4* | This study |
| pSP569 | pRS415-*SDH4* | [1] |
| pSP419 | pRS415-*CTH2* | [1] |
| pSP571 | pRS415-*CTH2-C190R* | [1] |
| pSP529 | pRS415-*GAL1-SDH4* | [1] |
| pRS416 | CEN *URA3* | [58] |
| pSP888 | pRS416-Flag_2_-*SDH4* | This study |
| pSP527 | pRS416-*SDH4* | [1] |
| pSP889 | pRS416-Flag_2_-*SDH4-AREmt* | This study |
| pSP528 | pRS416-*SDH4-AREmt* | [1] |
| pSP414 | pRS416-Flag_2_-*CTH2* | [1] |
| pSP410 | pRS416-*CTH2* | [1] |
| pSP760 | pRS416-Flag_2_-*CTH2*-*AREmt* | [13] |
| pSP758 | pRS416-*CTH2*-*AREmt* | [1] |
| pSP429 | pRS416-Flag_2_-*CTH2-C190R* | [13] |
| pSP427 | pRS416-*CTH2*-*C190R* | [1] |
| pSP457 | pRS416-*GFP*-*CTH2* | [10] |
| pSP724 | pRS416-*GFP-ΔN89-CTH2* | [10] |
| pSP464 | pRS416-*GFP-ΔN170-CTH2* | This study |
| pSP465 | pRS416-*GFP-ΔC52-CTH2* | This study |

58. Sikorski RS, Hieter P. A system of shuttle vectors and yeast host strains designed for efficient manipulation of DNA in *Saccharomyces cerevisiae*. Genetics. 1989;122: 19-27.
